# Modulation of Macrophage Immunometabolism: A New Approach to Fight Infections

**DOI:** 10.3389/fimmu.2022.780839

**Published:** 2022-01-26

**Authors:** Thierry Gauthier, Wanjun Chen

**Affiliations:** Mucosal Immunology Section, National Institute of Dental and Craniofacial Research (NIDCR), National Institutes of Health (NIH), Bethesda, MD, United States

**Keywords:** macrophage, immunometabolism, infections, SARS – CoV – 2, therapeutics

## Abstract

Macrophages are essential innate immune cells that contribute to host defense during infection. An important feature of macrophages is their ability to respond to extracellular cues and to adopt different phenotypes and functions in response to these stimuli. The evidence accumulated in the last decade has highlighted the crucial role of metabolic reprogramming during macrophage activation in infectious context. Thus, understanding and manipulation of macrophage immunometabolism during infection could be of interest to develop therapeutic strategies. In this review, we focus on 5 major metabolic pathways including glycolysis, pentose phosphate pathway, fatty acid oxidation and synthesis, tricarboxylic acid cycle and amino acid metabolism and discuss how they sustain and regulate macrophage immune function in response to parasitic, bacterial and viral infections as well as trained immunity. At the end, we assess whether some drugs including those used in clinic and in development can target macrophage immunometabolism for potential therapy during infection with an emphasis on SARS-CoV2 infection.

## Introduction

Macrophages are professional phagocytes patrolling most of the tissues, helping to maintain homeostasis and contributing to the first line of defense against pathogens (1). They are notably characterized by a high plasticity and ability to change their phenotype in response to different environmental stimuli (2). Macrophages derive either from an embryonic origin (deriving from the yolk-sac or the liver and maintained throughout the life by self-renewal) or originate from monocyte precursors (differentiating in the tissue after their infiltration) (3). Macrophages notably counter invading pathogens by recognizing defined pathogen associated molecular patterns (also known as PAMP) by a system of pathogen recognition receptors (PRR). Different classes of PRR have been so far described: the ALR (for Absent in melanoma 2 (AIM2)-like receptors) the CLR (for C-type lectin receptors), the NLR (for NOD-like receptors), the RLR (for RIG-I-like receptors), the TLR (for Toll-like receptors) and the cGAS (cyclic GMP–AMP Synthase)-STING (Stimulator of Interferon Genes) signaling (4, 5). ALR are composed of AIM2 and IFI16 (Interferon (IFN)-Inducible protein 16) which can sense cytosolic and nuclear DNA by assembling inflammasomes (6, 7). RLR are composed of RIG-I (Retinoic acid-inducible gene I), MDA-5 (Melanoma differentiation factor-5) and LGP-2 (Laboratory of genetics and physiology-2) which detect viral RNA and DNA. NOD1 (Nucleotide-binding oligomerization domain-containing protein 1) and 2 belong to the NLR and recognize gram positive and negative bacteria. TLR are the best described family of PRR and 10 members belong to this family in human (TLR1 to 10) and 12 in mice (TLR1 to 13 except TLR 10). They recognize a wide variety of PAMP including bacterial, parasitic and viral ligands (4, 8–10). Finally, the cGAS-STING pathway can recognize microbial and cytosolic DNA (11). While all these PRR trigger different molecular signaling, they will lead to the generation of an innate immune response *via* the production of pro-inflammatory molecules (cytokines, chemokines and DAMP (Damage Associated Molecular Patterns). The PRR recognizing viral PAMP will also trigger the secretion of type I interferon (IFN) which are crucial molecules in the antiviral response (4, 10, 12). These signaling cascades lead to the activation of different components of innate and adaptive immune responses, host cell metabolism and phagocytosis (13, 14). An important hallmark of macrophages is their plasticity in response to environmental cues. During bacterial and viral infections, the PRR stimulation and the pro-inflammatory micro-environment will enable macrophages to be activated toward a pro-inflammatory phenotype (also called classically activated macrophages or M1). On the other hand, a parasitic infection will result in the differentiation of alternatively activated macrophages, notably through the effects of IL-4 and IL-13 (also called anti-inflammatory phenotype, or M2). These different polarized states will help the macrophages to sustain their functions during homeostasis and in diseases, including infections (15–17). Notably, a growing body of evidence show that macrophages change their activation state through reprogramming of the metabolism. These metabolic changes, not only provide energy but also sustain changes in function and phenotype (18). In this review, we will highlight the main metabolic pathways and discuss how they regulate macrophages functions in response to different types of infections. We will also assess how the innate immune memory of macrophages during infections (called trained immunity) can be supported by changes in metabolism. Finally, we will envision the possibility of targeting macrophage immunometabolism as a possible therapeutic target to infections.

## Main Metabolic Pathways Used by Macrophages

**Figure 1:**
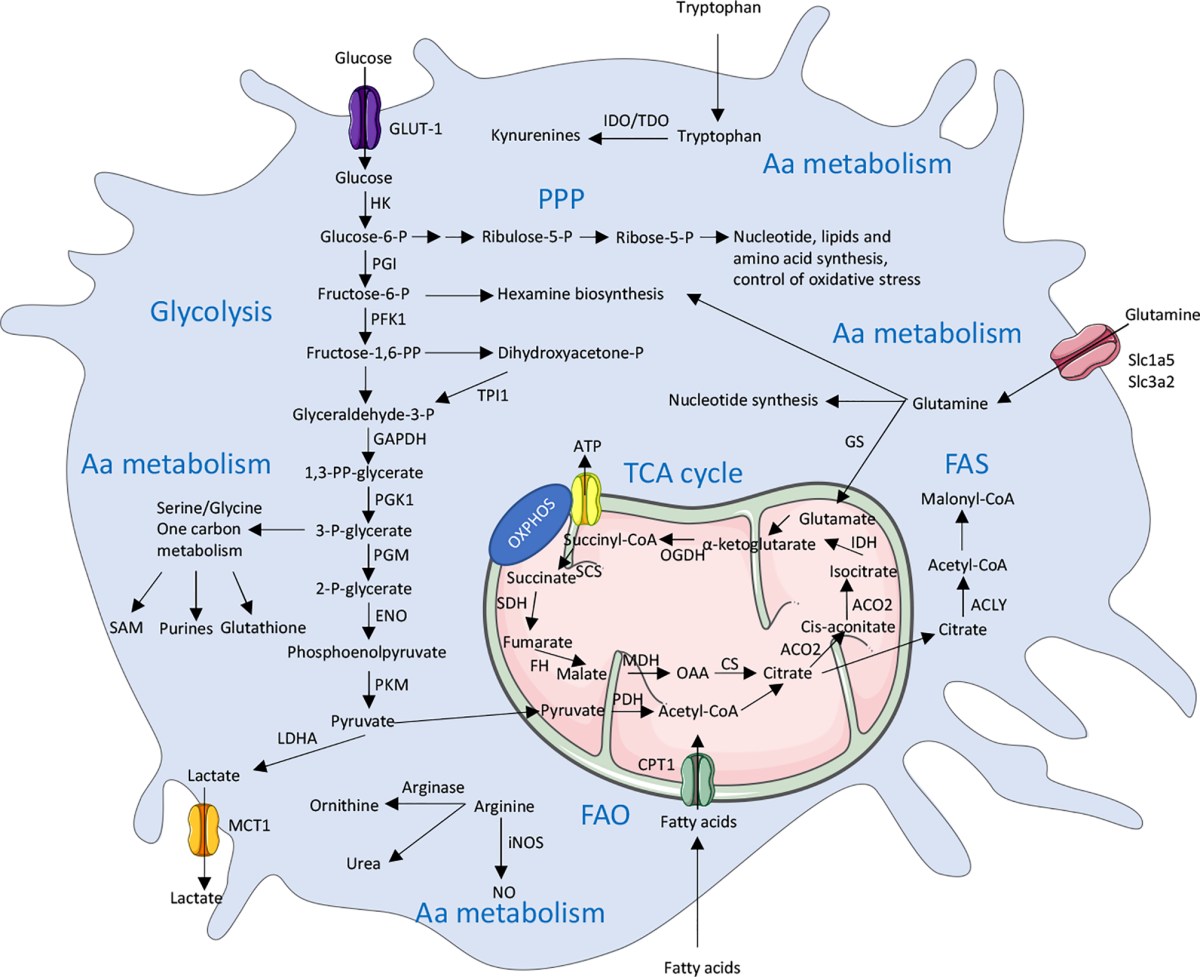
Overview of the main metabolic pathways used by macrophages. There are 5 major pathways used by macrophages to provide energy in cells including glycolysis, TCA (Tricarboxylic acid) cycle, PPP (Pentose phosphate pathway), FAS (Fatty acid synthesis) and FAO (Fatty acid oxidation) and amino acid (Aa) metabolism. These pathways are highly interconnected and are tightly regulated in immune cells, including macrophages. ACLY, ATP citrate lyase; ACO2, Aconitase 2; ATP, Adenosine triphosphate; CPT1, Carnitine palmitoyltransferase 1; CS, Citrate synthase; ENO, Enolase; FH, Fumarase; GAPDH, Glyceraldehyde 3-phosphate dehydrogenase; GLUT1, Glucose transporter 1; HK, Hexokinase; GS, Glutamine synthetase; IDH, Isocitrate dehydrogenase; IDO, Indoleamine 2,3-dioxygenase; LDHA, Lactate dehydrogenase; MCT1, Monocarboxylate transporter 1; MDH, Malate dehydrogenase; NO, Nitric oxide; iNOS, inducible NO synthase; OAA, Oxaloacetate; OGDH, α-ketoglutarate dehydrogenase; OXPHOS, Oxidative phosphorylation; P, Phosphate; PDH, Pyruvate dehydrogenase; PFK1,Phosphofructokinase 1; PGK1, Phosphoglycerate kinase 1; PGI, Phosphoglucoisomerase; PGM, Phosphoglycerate mutase; PKM, Pyruvate kinase muscle isotype; PP, bisphosphate; SAM, S-Adenosyl methionine; SCS, Succinyl coenzyme A synthetase; SDH, Succinate dehydrogenase; SLC, Solute carrier; TDO, Tryptophan 2,3-dioxygenase; TPI1, Triosephosphate isomerase 1.

Cell intrinsic metabolic changes are required in all cells to metabolize nutrients to help their survival, proliferation and differentiation. Five major pathways are used by macrophages to generate energy: i.e. the glycolysis, the tricarboxylic acid (TCA) cycle, the pentose-phosphate pathway (PPP), the fatty acid metabolism [including the fatty acid oxidation (FAO) and the fatty acid synthesis (FAS)] and the amino acid metabolism. In addition to generating energy, macrophages also produce intermediates metabolites that support their phenotype reprogramming in response to external stimuli. Interestingly, these diverse metabolic pathways are closely linked to each other and interconnected as described below. In most cells, glucose is the primary source of energy. Once entering the cells through its transporters, glucose is broken down by glycolysis. Along all these different steps, glycolysis can be diverted to provide metabolites for the PPP pathway or the generation of amino acids, but its primary fate will be to enter the TCA cycle and finally feed the OXPHOS to generate energy in the form of ATP. While it has been thought for decades that the purpose of metabolic pathways is to generate energy, it now appears that producing intermediates metabolites is also important for cellular and molecular signaling (**Figure 1**). We will discuss in more detail the different steps of these five major pathways in the next sections.

### The Glycolytic Pathway

In macrophages, glucose from the extracellular environment typically enters the cell through the glucose transporter GLUT1 (Glucose transporter 1) [encoded by the gene *Slc2a1 (*Solute Carrier 2a1)] to fulfill glycolysis (19, 20) (**Figure 1**). Glucose is further catalyzed by the Hexokinases (Hk1-4) which phosphorylates glucose into glucose-6-phosphate. The glucose-6-phosphate then enters the glycolysis (through the form of fructose-6-phosphate) or the PPP (which will be discussed below). Fructose-6-phosphate can also be used by the phosphofructokinases (Pfkl, m, p) into glycolysis or diverted toward the hexosamine biosynthesis pathway. This pathway will lead to the generation of UDP-GlcNAc that is the substrate used for the glycosylation reactions (O- and N-GlcNAcylation). A downstream metabolite of glycolysis, the glyceraldehyde-3-phosphate can also lead to the generation of glycerol-3-phosphate and the biosynthesis of diverse lipids. Another possible break into the glycolysis is to enter the serine and glycine pathway from 3-phosphoglycerate. Serine can further be converted into folate to generate one-carbon units. The final glycolytic enzyme is the pyruvate kinase (PKM1 and 2 are the main isoforms in most tissues) which catalyzes the conversion of phosphoenolpyruvate into pyruvate. Pyruvate is then converted into 2 major metabolites. The first one is lactate which is generated by the lactate dehydrogenase (LDHA and B), and finally exported to outside the cell, in a process that is called aerobic glycolysis. While this process was originally described to occur in cancer cells due to a defect in mitochondria, this aerobic glycolysis clearly occurs during normal cellular processes in immune cells including macrophages. It appears that, despite generating less adenosine triphosphate (ATP) per molecule of glucose used, this mechanism can sustain a rapid activation of immune cells and preserve the redox balance through a tight control of the NADH levels. The second pathway is for the pyruvate to be oxidized in the mitochondria by the pyruvate dehydrogenase (PDH) which convert it into acetyl-coA to enter the TCA cycle (21–23).

### The TCA Cycle

The TCA cycle (also called Krebs cycle or citrate cycle) occurs into the mitochondria (**Figure 1**). It is initiated with the generation of acetyl-coA coming from three possible sources: the pyruvate from glycolysis, the fatty acyl-coA from fatty acids and the acetate (either coming from acetate metabolism or extracellular uptake). The acetyl-coA will, in combination with oxaloacetate, generate citrate which will be further oxidized into the TCA cycle. Citrate can also be exported to the cytosol to generate itaconate or to be hydrolyzed by ATP-citrate lyase (ACLY) in cytosolic acetyl-coA which will fuel the fatty acid and cholesterol synthesis (for the generation of new membranes) or will contribute to protein acetylation (notably histone acetylation) (24). Interestingly, cytosolic citrate can also exert a negative feedback on glycolysis by inhibiting, directly or indirectly, PFK and HK enzymes (25). The major products generated by the TCA cycle are NADH and FADH_2_ which can be transferred into the electron transport chain to support the oxidative phosphorylation (OXPHOS) and the efficient generation of ATP (26).

### The Pentose Phosphate Pathway

The glucose-6-phosphate (G6P) generated by hexokinases can be metabolized to enter the glycolysis or be directed into the PPP (which occurs in the cytosol) (**Figure 1**). The PPP is divided into 2 phases; an oxidative phase which will give raise to the reduction of NADP^+^ (Nicotinamide adenine dinucleotide phosphate) into NADPH linked to the conversion of G6P into ribulose-5-phosphate (R5P); a non-oxidative phase will generate ribose-5-phosphate. NADPH is an essential cofactor for the generation of antioxidants, ROS and NO, but also to generate lipids and nucleotides. The R5P is a precursor of nucleotides and amino acids synthesis (27). NADPH can further be used by the Fatty acid synthase to promote the generation of fatty acids or by the enzyme NADPH oxidase 2 (NOX2) to generate reactive oxygen species (ROS) ultimately leading to an oxidative burst (28, 29).

### The Fatty Acid Metabolism

Fatty acid Oxidation (FAO) is the most efficient producer of energy for the cell since a single molecule of fatty acid can generate as much as 100 molecules of ATP. The short chain fatty acids can passively enter the mitochondria, while the medium and long chain fatty acids need to be imported by the ligation to coA, which is then exchanged by carnitine palmitoyltransferase 1A (CPT-1A) upon mitochondrial transfer (**Figure 1**). The carnitine conjugated to the fatty acid is then shuttled into the mitochondria and the carnitine is removed by the CPT2 to give a molecule of fatty acid acyl-coA. The oxidation of this fatty acid will lead to produce large amounts of acetyl-coA, NADH and FADH2 which are used to augment the TCA cycle and the OXPHOS to generate ATP.

Fatty acid synthesis (FAS), on the other hand, uses precursors from the other metabolic pathways (glycolysis, TCA cycle and PPP) to generate lipids. Notably, the acetyl-coA is transformed into malonyl-coA by the acetyl-coA carboxylases. Seven molecules of malonyl-coA are then condensated to generate palmitate (the initial product of fatty acid synthesis) by the enzyme Fatty Acid Synthase. Palmitate, a 16 carbons saturated molecule, is then elongated and desaturated to generate fatty acid of diverse size and degrees of saturation (30, 31).

### The Amino Acid Metabolism

Amino acids availability is crucial for multiple aspects of cell biological functions. Since there is a large number of different amino acids, there are different pathways leading to the utilization and generation of amino acids. They can be divided into two categories: the essential amino acids which cannot be synthesized by the human body (and therefore need to be taken from nutrition) and the non-essential amin acids which can be synthesized by the body (32).

An important amino acid for the macrophage behavior is glutamine. Glutamine enters the cell through a diverse range of Slc transporter including Slc1a5 (Solute carrier 1a5) and Slc3a2, which are highly expressed in macrophages (33) (**Figure 1**). Glutamine can then contribute to the generation of nucleotides or UDP-GlcNac or enter the mitochondria to generate glutamate (34). The glutamate can generate glutathione (which can help to control the redox balance) or be converted into α-ketoglutarate to enter the TCA cycle. The glutamate is also a donor for the generation of many different amino acids (35).

Serine is a central hub for cell metabolism. As described previously, it can be converted from the glycolytic metabolite 3-PG (3-phosphoglycerate). The conversion of Serine to Glycine is an outcome of Serine generation which can later lead to production of glutathione. Serine is also a major source for the one-carbon metabolism pathway which will serve as a building block for S-adenosylmethionine (and the regulation of protein methylation), nucleotides, NAD(P)H, and ATP. Finally, this pathway can also fuel the folate metabolism leading to the production of purines (36).

Another important amino acid in term of immunometabolism is Arginine. Arginine can be produced by many different pathways (including extracellular uptake and intracellular production) to support cell growth and proliferation (37). An important feature, in macrophages, is the ability of arginine to be catalyzed either by NOS (Nitric Oxide Synthase) to generate NO (Nitric oxide) and citrulline or to be catalyzed by Arginase 1 (Arg1) to ornithine and urea (38). Of note, Arg1 has been long described to be expressed by anti-inflammatory M2 macrophages while the expression of iNOS has been demonstrated to be a marker of pro-inflammatory M1 macrophages (38).

L-tryptophan is also an essential amino acid coming from dietary intake. A small fraction of tryptophan is used to the production of proteins and neurotransmitters; however, the major part is used to fuel the kynurenine pathway which give raise to several metabolites. The first step of this reaction is the conversion of tryptophan into N-formylkynurenine, which is catalyzed by the rate-limiting enzymes IDO1,2 (Indoleamine-2,3-dioxygenase 1 and 2) and TDO (Tryptophan-2,3-dioxygenase) (39, 40). Interestingly, it appears that the tryptophan metabolism in macrophages promotes immune tolerance by increasing the generation of M2 macrophages and by depleting extracellular tryptophan, thus modulating T cell functions (41, 42).

While all these metabolic pathways are described as distinct entities, they are highly interdependent and inter-regulated demonstrating a tight and complex regulation of cellular metabolism. A good example of this complex regulation is the kinase serine/threonine kinase mTOR (mammalian Target of Rapamycin). mTOR is composed of 2 different complexes mTORC1 and 2 (mTOR Complex). mTOR activation has been widely demonstrated to be regulated by the level of amino acid in the cell but mTOR is also regulated by the levels of glucose, oxygen and DNA damage (43, 44). Downstream, mTOR can regulate lipid synthesis or the PPP through SREBP (sterol responsive element binding protein) and the glycolysis through the transcription factor Hif-1α (Hypoxia induced factor 1 alpha), both of which can transcriptionally activate genes that encode enzymes belonging to these pathways (45, 46).

Importantly, these different pathways are used by all mammalian cells to generate energy and intermediate metabolites. However, different cells can modulate the use of these pathways to adapt their function, development, or proliferation. In the context of macrophage immunology, it appears that, despite the fact they are long-lived non proliferative cells, pro- and anti-inflammatory macrophages use different pathways to meet their needs, differentiate into M1 and M2 macrophages and perform their function. More specifically, it has now been demonstrated that the pro-inflammatory M1 macrophages rely on an increased dependency on glycolysis and PPP, while they remodel largely their TCA cycle and depend less on OXPHOS to generate energy. This will allow them to sustain an inflammatory phenotype (increased phagocytosis, production of pro-inflammatory cytokines and chemokines, NO and ROS production, and enhanced bacterial killing). On the other hand, the anti-inflammatory M2 macrophages will use the TCA cycle, the FAO and the OXPHOS to generate energy, while relying less on glycolysis. They will also promote the glutamine metabolism and arginase activity. This will promote the expression of M2 markers, the production of anti-inflammatory cytokines and their pro-repair functions. Interestingly, it appears that other pro- or anti-inflammatory immune cells (for example Th1/TH17 versus Treg) use similar pathways than macrophages to sustain their pro- (glycolysis, PPP) and anti-inflammatory (TCA cycle, OXHPOS, FAO) phenotypes. This highlights the crucial role of the microenvironmental cues to modulate immune cell metabolism and functions (18, 47). We will now describe in details how macrophages adapt their metabolic responses during different types of infections.

## Macrophage Immunometabolism in Infections

### Role of Macrophage Immunometabolism During Parasitic Infection

Parasitic infections such as helminths and protozoans represent a major health concern in developing countries. According to the US Center for Disease Control and Prevention (CDC), malaria (caused by a protozoan named *Plasmodium)* is responsible for the death of 600 000 people each year, mainly in sub-Saharan Africa. Helminths, in the other side, could infect up to 1.5 billion people worldwide and lead to diverse manifestations like diarrhea, respiratory symptoms, asthma-like symptoms, as well as neurologic and motor disorders (16, 48, 49).

### Macrophage Metabolism in Helminth Infection and IL-4 Dependent Polarization

**Figure 2:**
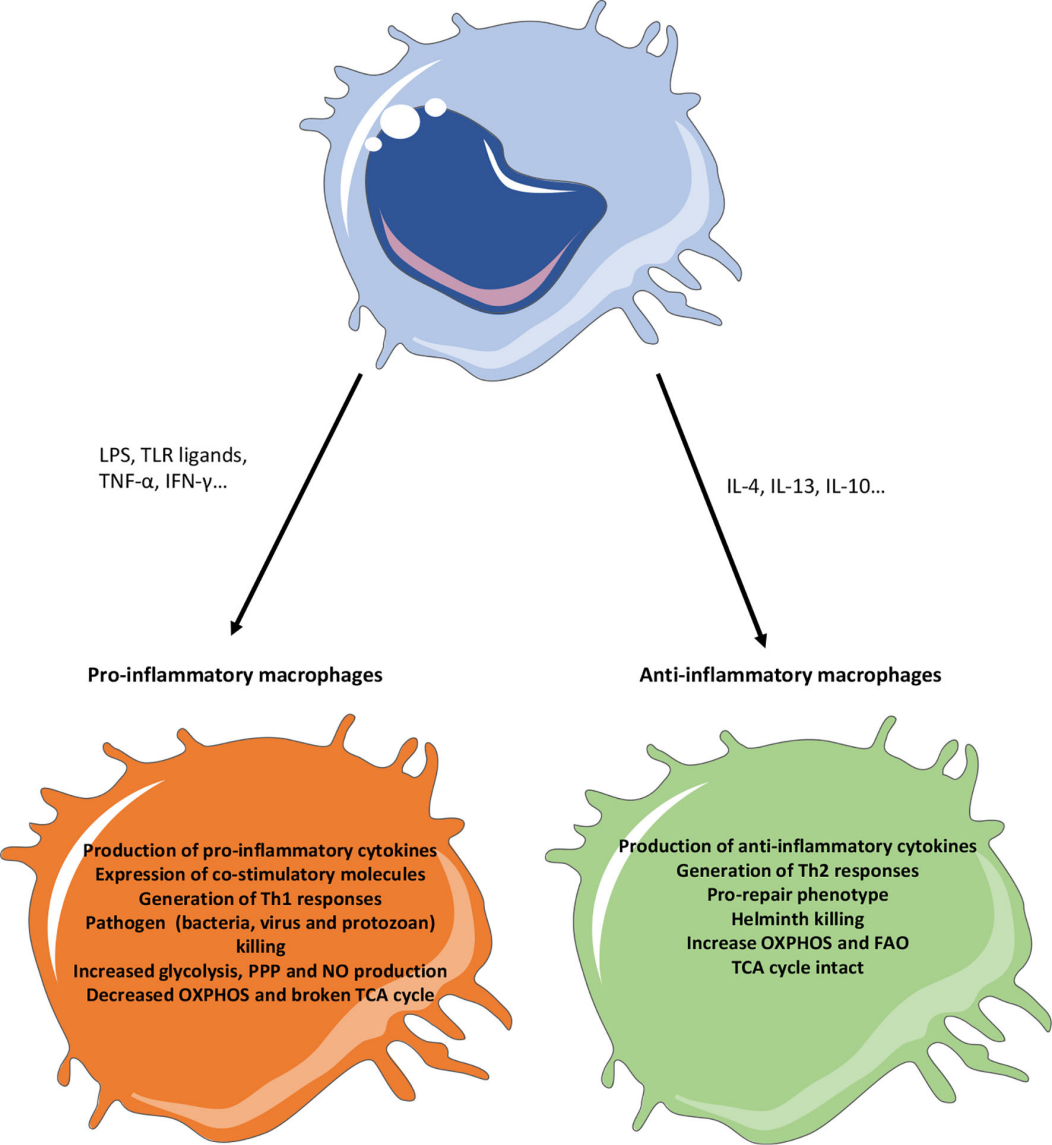
Phenotypic characteristics of pro- versus anti-inflammatory macrophages. Pro-inflammatory stimuli (like TLR ligands or pro-inflammatory cytokines) will generate a pro-inflammatory response in macrophages, notably characterized by the production of pro-inflammatory cytokines, the expression of co-stimulatory molecules and a Th1 response. On the other hand, anti-inflammatory stimuli (like IL4, IL13 or IL10) will promote a pro-repair phenotype in macrophages notably caracterized by the production of anti-inflammatory and pro-resolutive factors and the generation of a Th2 response. In the context of infection, the generation of pro-inflammatory macrophages will promote their killing activity but microbes will try to promote the generation of anti-inflammatory phenotype to escape these responses. Anti-inflammatory macrophages, while promoting infections in general, will have a strong anti-helminth effect. Metabolically, the pro-inflammatory macrophages use glycolysis and PPP to produce energy and have a broken TCA cycle. Instead, anti-inflammatory macrophages use the FAO and OXPHOS to provide cellular energy. FAO, Fatty acid oxidation; IFN, Interferon; IL, Interleukin; LPS, Lipopolysaccharide; OXPHOS, Oxidative phosphorylation; NO, Nitric oxide; PPP, Pentose phosphate pathway; TCA, Tricarboxylic acid; Th, T helper; TLR, Toll like receptor; TNFα, Tumor necrosis factor alpha.

Helminths generate a type 2 immune response in the infected organs, inducing the release of high levels of IL-4 and IL-13, which will instruct the macrophages to adopt an alternatively activated macrophages (50, 51). IL4 and IL13 can be produced by T cells, innate lymphoid cells (ILC), basophils and eosinophils. AAM express typical markers like RELMα (Resistin-like molecule-alpha), VEGF (Vascular Endothelial Growth Factor), Arg1, YM1, IGF-1 (Insulin-like Growth Factor 1) or TGF-β (Transforming Growth Factor Beta) which will help them to control parasite confinement in granulomas and their clearance, as well as tissue repair and control of the immune response. In addition, the transcription factors associated with this AAM phenotype include STAT6 (Signal Transducer and Activator of Transcription 6), GATA3 (GATA binding protein 3) or PPARγ (Peroxisome Proliferator-Activated Receptor gamma) (50, 52) (**Figure 2**).

The up-regulation of FAO is a crucial feature of AAM (53, 54). This is orchestrated by a STAT6-PPARγ/PGC-1β signaling pathway, finally leading to the expression of specific markers of AAM and their survival (53, 55, 56). The main source of fatty acids for IL4 treated macrophages is through uptake of fatty acids *via* the scavenger receptor CD36 or through the lysosomal lipolysis *via* lysosomal acid lipase, which both sustain the expression of alternative markers (57). Interestingly, during *H. polygyrus* infection, the inhibition of lipolysis block AAM differentiation and the elimination of the parasite in an IL4 setting (57). However, two recent publications challenged the previous findings demonstrating that FAO is indispensable for AAM polarization. These publications demonstrated that the genetic depletion of Cpt1a and Cpt2 does not inhibit the IL4 induced polarization (58, 59). They also observed that the widely used FAO inhibitor etomoxir, at the doses commonly used to inhibit FAO, inhibits IL4 polarization by targeting the CoA metabolism instead of the FAO (59). In fact, despite its effect observed at low-dose (FAO inhibitor), a high dose of etomoxir can disrupt intracellular CoA homeostasis therefore leading to block the IL4-induced polarization. The example of etomoxir is of great interest for the field of immunometabolism. In fact, many of the findings in this field rely on the use of inhibitor, some of which could have several off-targets, and tightly controlling the dose used when performing experiments appears to be of crucial importance. This also highlights the necessity to confirm the findings observed by using inhibitors with other techniques like gene knock-down and to proceed carefully in the interpretation of the results. The alternatively activated macrophages also increase glycolysis in response to IL4 in a manner dependent on an AKT-mTORC2-IRF4 (Interferon regulatory factor 4) signaling pathway (60, 61). Interestingly, the loss of mTORC2 in macrophages during helminth infection by *H. polygyrus* (*Heligmosomoides polygyrus*) lead to lose the AAM polarization and their ability to clear the infection (61). A possible outcome for this increased glycolysis is to feed, together with glutamine, the hexosamine biosynthetic pathway to promote protein glycosylation. In fact, the inhibition of this pathway with tunicamycin (an inhibitor of N-glycosylation) prevent the expression of some AAM markers (54). Another outcome of this increased glycolysis is possibly to fuel lipid synthesis, acetyl-coA production and TCA cycle (54, 62). The pool of acetyl-coA (notably coming from the cleavage of cytosolic citrate from the enzyme ACLY which is activated in an AKT-mTORC1 pathway) is used by macrophage in this context to promote the histone acetylation of IL-4 inducible genes (60). However, some recent publications suggest that the link between glycolysis and AAM might be more complex. This question has been raised because some publications demonstrated that some inhibitors used to study the role of glycolysis (ACLY inhibitor and 2-DG) have a broader effect than just inhibit their primary target (63). In fact, glucose depletion or galactose treatment, while affecting glycolysis, does not affect the AAM polarization. In the meantime, 2-DG, which can suppress both glycolysis and AAM polarization, likely affect the AAM polarization by modulating the ATP levels and the JAK-STAT6 signaling (64). Thus, several questions about the role of glycolysis during IL-4 polarization and helminth infection remain unanswered. Firstly, how the glycolysis pathway is up-regulated during AAM; secondly, what is the exact role of glycolysis during AAM polarization and how it affects helminth infection; and finally, which metabolic pathway the different glycolytic metabolites are preferentially fueling.

In the meantime, IL4 activation limits the use of the PPP in macrophages by increasing the expression of the sedoheptulose kinase CARKL (Carbohydrate kinase-like) that limits the production of sedoheptulose-7-phosphate, thus promoting an alternative activation (65). However, the mechanisms by which the PPP interacts with the IL-4 polarization is not fully understood and needs to be further investigated.

IL4 treated macrophages generate ATP through an oxidative TCA cycle coupled to OXPHOS (54) (**Table 1**). To fuel the TCA cycle, AAM will use the glutaminolysis or the FAO. The degradation of glutamine through glutaminolysis in IL-4 treated macrophages will generate α-ketoglutarate which will promote the AAM polarization through 3 different mechanisms: 1) it is used to fuel the FAO; 2) it induces an epigenetic reprogramming demethylation of H3K27 on the promoters of AAM-specific genes; 3) it favors PHD activity leading to the inhibition of NFkB pathway (66).

**Table 1:** Metabolic changes induced during pathogen infections.

Pathogen	Helminth	Protozoa	Bacteria	Virus	Trained immunity
**Glycolysis**	Increased glycolysis (possibly to feed the TCA cycle or the Hexosamine pathway).	Depending on the pathogen: *L. infantum* increases glycolysis while *L. donovani* and *L. amazonensis* don’t. Support the clearance of *T. cruzi.*	Increased glycolysis levels. Increased expression and/or activation of most glycolytic genes (GLUT1, HK1/2, GAPDH, PKM2…) which promotes the production of pro-inflammatory cytokines (HMGB1, IL1β, IL6, TNFα…).	Role is dependent on viral infection and timeline. Protective during RSV infection and HIV-1 but detrimental during norovirus and HIV-1 infections.	Increased glycolysis through AKT-mTOR-HIF1α.
**PPP**	Limited use of PPP through overexpression of CARKL.	Support the clearance of *T. cruzi*.	Increased PPP (notably through dowregulation of CARKL) which support the inflammation.	Role largely unknown. Might be decreased during HIV-1 infection.	Role unknown.
**FAO**	Up-regulation of FAO and lysosomal lipolysis. FAO feed the TCA cycle.	Increased during *T. cruzi* infection	Role unknown.	Cholesterol and FA import are increased which promote infection during HIV or MHV-68 infections.	Role unknown.
**FAS**	Role unknown.	Increased during *T. cruzi* infection	Possibly increased to sustain the inflammasome activation and IL1β/IL18 production.	Increased production of MUFA during TLR7/9 stimulation (decrease during TLR3) which controls the expression of pro-inflammatory genes.	Role unknown.
**TCA cycle/OXPHOS**	TCA cycle intact and OXPHOS increased to generate energy.	Depending on the pathogen: *L. infantum* switch from OXPHOS to glycolysis while *L. donovani* and *L. amazonensis* promote OXPHOS.	TCA cycle broken. Increase in citrate (which fuel PGE2, ROS and NO production; also activates ACLY which promote LPS-induced gene expression), increase in itaconate (which inhibits bacterial growth but limit inflammation) and increase in succinate (which stabilize HIF1α and promote pro-inflammatory gene expression).	Altered TCA cycle and OXPHOS during HIV infection.	Decreased OXPHOS.
**Aa metabolism**	Glutamine: feed the TCA cycle, promote anti-inflammatory gene expression and inhibit the NFkB pathway. Arginine: Arg1 expression highly increased. Tryptophan: expression of IDO decreased and depletion of tryptophan. Lamtor1 is critical for expression of IL4 induced markers.	Arginine is depleted by macrophages to prevent pathogen growth during *Leishmania* infections.	Glutamine is crucial for the production of NO and IL-1β through feeding of the TCA cycle. Serine is also crucial for the production of IL1β. The arginine metabolism is crucial for anti-bacterial response (notably *via* the production of NO). The role of tryptophan is still unclear.	Glutamine is a crucial source of energy during HIV latent infection and has detrimental effect. IDO expression is increased during HIV and EBV infections and its blockade lead to kill infected macrophages. Role of Arginine metabolism is depending of the phase infection and can be beneficial or detrimental. mTOR is largely modulated by viruses to promote cellular infection.	Glutaminolysis is required for the induction of trained immunity through control of HIF-1α/KDM5 induction of TNFα and IL-6. The role of other Aa remains unknown.

Aa, Amino acid; ACLY, ATP-citrate lyase; Arg1, Arginse 1; CARKL, Carbohydrate kinase-like; EBV, Epstein-Barr virus; FAO, Fatty acid oxidation; FAS, Fatty acid synthesis; GAPDH, Glyceraldehyde 3-phosphate dehydrogenase; GLUT1, Glucose transporter 1; HIV, Human immunodeficiency virus; HIF1α, Hypoxia factor 1 alpha; HMGB1, High–mobility group box 1; HK, Hexokinase; IDO, Indoleamine 2,3-dioxygenase; IL, Interleukin; KDM5, Lysine deacetylase 5; Lamtor1, Late endosomal/lysosomal adaptor and MAPK and mTOR activator 1; MHV-68, Murine gammaherpesvirus-68; MUFA, Monounsaturated long chain fatty acid; mTOR, mammalian target of rapamycin; NFkB, Nuclear factor kappa B; NO, Nitric oxide; OXPHOS, Oxidative phosphorylation; PGE2, Prostaglandin E2; PKM, Pyruvate kinase muscle isotype; PPP, Pentose phosphate pathway; ROS, Reactive oxygen species; RSV, Respiratory syncytial virus; TCA, Tricarboxylic acid; TNFα, Tumor necrosis factor alpha.

A crucial regulator of AAM polarization is the protein LAMTOR1 (Late endosomal/lysosomal adaptor and MAPK and mTOR activator 1). LAMTOR1 is a component of the mTORC1 complex which is necessary for the recruitment of mTORC1 to the lysosome in response to amino acid stimulation (67). Importantly, macrophages deficient for LAMTOR1 or depleted in amino acids in the media are completely unable to express the main IL-4 induced markers (e.g., Arginase I, Mannose receptor, IL10 and RELMα) demonstrating a decisive role of amino acids for the polarization (68). IL4 treated macrophages metabolize arginine to urea and ornithine *via* an increased expression of the Arg1 (69). Arg1 expression is induced by a STAT6-Cebp/β (CCAAT-enhancer-binding proteins beta) (70, 71). While Arg1 is one of the most used markers to define AAM, its exact role in macrophage polarization remains largely unexplored. Some studies suggest that, notably through the synthesis of polyamines, Arg1 might promote tissue repair during infections (72, 73). Downstream of arginine, the polyamine-eIF5-hypusine pathway regulates IL4 mediated polarization of macrophages and the blockade of this pathway inhibits the protective effect of IL4 during *H. polygyrus* infection (74). On the other hand, the resistance to infection by the helminth *Trichuris muris* is unaffected by the deletion of Arg1 in macrophages suggesting more complex roles of arginine metabolism during helminth infection. In this context, macrophages treated by IL4 downregulate the expression of IDO and promote the expression of the immunoregulatory phenylalanine oxidase IL4L1 (IL4-induced gene 1) leading to the depletion of tryptophan (75, 76). However, the consequence of this depletion is largely unexplored. One suggestion is that the depletion of tryptophan in the micro-environment remove a source of energy used by helminths.

### Macrophage Metabolism During Protozoan Infection

Macrophages also play a crucial role in the immune responses to protozoan infections. During protozoan infections, macrophage polarization toward a pro-inflammatory phenotype will play a role in the clearance of the pathogen. Macrophages will use the respiratory burst and production of ROS (Reactive oxygen species), NO (Nitric oxide) and pro-inflammatory cytokines (TNFα, IL6, IFNγ) as ways to kill the pathogen and initiate an adaptive immune response if needed (77). Macrophages are responsible for the destruction of the parasites, yet paradoxically also provide a way for parasite to replicate. In fact, protozoan could polarize macrophages toward an anti-inflammatory phenotype (similar to the one induced by IL4 during helminth infection) to escape the killing by macrophages and favor their replication (78). Notably, the expression of Arg1, Mannose receptor (also called CD206) or PPARγ in macrophages are detrimental to the host response during Leishmania infections (79–81).

At basal state, macrophage infection with *L. donovani* and *L. amazonensis* increases the OXPHOS levels, without affecting the glycolysis, which is linked to an increase in the production of pro-inflammatory cytokines and chemokines (82) (**Table 1**). During *L. infantum* infection, macrophages transiently increase aerobic glycolysis which is followed by a later sustained increased in OXPHOS through a SIRT1 (Sirtuin 1)-LKB1 (Liver kinase B1)-AMPK (AMP-activated protein kinase) pathway. In this context, the deletion of SIRT1 or AMPK in mice led to promote parasite clearance (83). Interestingly, arginine, which is a crucial metabolite for the growth of *Leishmania* parasite, is depleted by macrophages (either through NO or polyamines). To counterbalance the depletion of arginine in infected macrophages, *Leishmania* induces the overexpression of many arginine transporter. These findings demonstrate that the interaction between host and pathogen metabolism is crucial to control the infection (84). During *Trypanosoma cruzi* infection, the glycolysis and OXPHOS do not appear to be modulated in macrophages (82). However, FAO and lipids production are increased, and they promote the pathogen replication. This appears to be dependent on the ability of T. cruzi to promote the expression of LDLR (Low Density Lipoprotein Receptor) therefore leading to the accumulation of LDL and cholesterol into the cells. However, the exact downstream mechanisms remain to be elucidated (85, 86). Interestingly, IFN-γ treatment of macrophages infected with *T. cruzi* support the up-regulation of a glycolysis-PPP axis important for the production of ROS and NO and the clearance of the pathogen (87). *T. brucei* produces large amounts of indolepyruvate (a transamination product of tryptophan). This metabolite reduces the host level of HIF1α and the production of IL1β as well as the glycolysis during LPS-induced inflammation (88). While the links between helminth infection and macrophages start to be understood (mostly due to the study of IL4 treated macrophages), further mechanistic studies will be necessary to decipher how macrophages modulate their metabolism to fight protozoa infection compared to how the pathogen modulate their metabolism to favor its survival.

## Role of Macrophage Immunometabolism During Bacterial Infection

During encounter of bacteria, macrophages are able to sense the pathogen through the system of PAMP-PRR. A well described PAMP is LPS that signals through TLR4. These stimuli will generate a pro-inflammatory phenotype of macrophages typically characterized by their ability to kill pathogens and elicit an adaptive immune response *via* antigen presentation. The macrophages express high levels of co-stimulatory molecules like CD40 (Cluster of differentiation 40), CD80, CD86, as well as MHC-II (Major Histocompatibility Complex II) to perform antigen presentation. They also produce pro-inflammatory cytokines such as TNFα, IL6, IL1β, IL12 and IL23 which will promote a TH1 (T helper 1) response leading to the production of IFNγ. The production of these cytokines will also further polarize the macrophages toward a more pro-inflammatory profile through a positive feedback loop. Th1 cells, through production of IFNγ, will reinforce this pro-inflammatory polarization of macrophages notably by enhancing their ability to clear the bacteria (via increased phagocytosis, autophagy, phagolysosomal maturation and promoting cytokines production). In the meantime, the enhanced production of NO and ROS will also provide mechanisms to enhance bacterial clearance. The expression of these factors is controlled by a network of transcription factors including NFkB (Nuclear factor kappa B), STAT1 and 3, HIF1α or the IRFs (15, 23, 89). While the principle of immunometabolism has been documented in many different contexts, the most well studied one is during the bacterial infections associated with LPS stimulation (coupled or not to IFNγ stimuli) (**Table 1**).

It has been shown that macrophages treated by LPS+IFNγ largely up-regulate their aerobic glycolysis to provide energy to the cell in a more efficient way (54). This increase is mediated by the glucose transporter GLUT1 which is up-regulated after bacterial stimuli. Interestingly, the deletion or up-regulation of Slc2a1 result in changes in the expression of many inflammatory genes (for example nos2, serpine1, mcp-1) (19, 20). The first step of glycolysis is the generation of glucose-6-phosphate by the Hexokinases (and mostly in immune cells by HK1 and HK2). HK1 is regulated by mTORC1 and HK1-induced glycolysis is necessary for the activation of the inflammasome (90). HK2 exerts a similar effect through its localization in the mitochondrial membrane. The release of HK2 from the outer membrane of the mitochondria is a sufficient event to trigger the activation of the NLRP3 inflammasome and the IL1β/IL18 production (91). The rate-limiting enzyme PFKL has been identified as a negative regulator of the oxidative burst in the context of *Staphylococcus aureus* infection. When PFKL is deleted, glucose is diverted in the PPP rather than entering glycolysis and sustains the production of NADPH finally leading to enhanced bactericidal activity through an unregulated respiratory burst (92). Another glycolytic enzyme, aldolase can also play a role in macrophage immunometabolism. In fact, treatment of macrophages with itaconate, a well-known anti-bacterial product which is a by-product of citrate and TCA activity, induces the inhibition of aldolase during LPS stimulation and prevents the production of IL1β demonstrating that aldolase promotes the production of IL1β (93). Downstream of aldolase, LPS can also regulate GAPDH through the malonylation of its lysine 213 which will regulate its activity and its binding to the TNFα mRNA, leading to an enhance translation and cytokine production (94). Moreover, macrophage treated with 4-Octyl itaconate modulates GAPDH activity and glycolysis leading to a decrease in LPS-induced inflammation *in vitro* and in a sepsis model (95). Another article linked GAPDH to an anti-inflammatory response in mice. In fact, treatment of LPS-induced sepsis mice with GAPDH lead to decrease inflammation and improve survival in a not fully understood mechanism suggesting that the regulation of GAPDH might be a tight point of control for the inflammatory response. However, in this study, the authors injected high levels (10 mg/kg) of GAPDH originated from rabbit muscle in a systemic manner suggesting that this effect might not be relevant to decipher the physiological role of GAPDH in inflammation (96). α-enolase, which catalyzes the conversion of 2-phosphoglycerate into phosphoenolpyruvate has also been described to be expressed at the surface of monocytes and macrophages from rheumatoid arthritis patients and a mouse model of arthritis. The activation of surface α-enolase trigger the production of inflammatory factors (TNFα, IL1β, IFNγ and PGE2) and could be detrimental for the pathology but could be beneficial during bacterial infection (97). Finally, PKM2, the major isoform of pyruvate kinases expressed in macrophages, is also up-regulated after LPS treatment. Interestingly, the activity of PKM2 is also regulated by its ability to dimerize or tetramerize (while other PK isoforms only exists as tetramers). PKM2 tetramer acts as a pyruvate kinase enzyme and therefore it regulates glycolysis, while the PKM2 dimer can exist in different localizations and has a moonlighting function (it can notably regulates the mitochondria and ER functions or regulates gene expression in the nucleus) (98). LPS induces the tetramerization of PKM2 leading to decrease its nuclear localization and the expression of glycolytic and HIF-1α-induced genes. However, its dimerization promotes the expression of pro-inflammatory genes. These findings are also applicable *in vivo* since the activation of PKM2 decreases the inflammation and the bacterial load in a model of LPS-sepsis and a model of *Salmonella typhimurium* infection (99). The knockdown or inhibition of PKM2 confirmed that PKM2 is crucial for the inflammatory effect of LPS since it also inhibits the NLRP3 and AIM2 inflammasomes activation, the HMGB1 (High–mobility group box 1) release and improves the survival of the mice in a model of LPS-induced septic shock (100, 101). Moreover, the inhibition of LDHA (the final step enzyme of glycolysis) might also protect cells against LPS induction of pro-inflammatory genes (102). Finally, most of the glycolytic enzymes are overexpressed after LPS treatment in a HIF-1α-dependent manner, suggesting the regulation of immune responses through glycolytic enzymes might be much wider than previously thought and might also be tightly regulated by the network of transcription factors expressed (47, 103). However, many of the findings obtained to prove the role of glycolysis in supporting inflammatory functions of LPS-treated macrophages rely on the use of inhibitors that may possibly be non-specific (e.g., 2-DG as it has been described before) or on the deletion of transcription factors playing a broad role in the generation of proper immune response (e.g., HIF1α). Thus, it needs more studies and development of specific tools to target glycolysis to fully understand the role of glycolysis in the development of anti-bacterial responses.

The increase in glycolysis in these cells is substantially rerouted toward the PPP by downregulating the inhibitory sedoheptulose kinase CARKL. This increase in PPP in turn supports pro-inflammatory macrophage phenotype. It might promote NADPH production necessary for the NADPH oxidase and iNOS activity thus supporting an antibacterial function (65, 104).

While IL4 treated macrophages are not link to changes in the TCA cycle, LPS-treated macrophages largely remodel their TCA cycle through breaks at several key points of the cycle leading to accumulation of citrate, succinate and itaconate (54, 104, 105). Accumulation of citrate is due to a decreased expression of IDH (Isocitrate dehydrogenase) and increased expression of CIC (Citrate carrier) (leading to its removal from the mitochondria) (54, 106). Citrate can then be utilized to fuel the production of PGE2 (Prostaglandin E2), NO and ROS (through increased FAS and NADPH production) (106). Another crucial role of citrate is to promote histone acetylation *via* ACLY and the expression of LPS responsive genes (105–108). The next break occurs at the level of itaconate. Itaconate production is enhanced because of the increased expression of IRG1 (Immune-Responsive Gene 1) (54, 109). Itaconate inhibits directly the growth of bacteria like *Salmonella enterica* and *Mycobacterium tuberculosis* (*Mtb*) by targeting the isocitrate lyase demonstrating a strong anti-bacterial effect (109). Despite this effect, itaconate has an anti-inflammatory effect on macrophage activation triggered by LPS+IFNγ treatment by limiting the production of pro-inflammatory factors including IL1β, IL6, IL12, NO or HIF1α. This occurs through different mechanisms that include the regulation of succinate oxidation (and the level of OXPHOS), the activation of a KEAP1 (Kelch-like ECH-associated protein 1)-NRF2 (Nuclear factor erythroid 2-related factor 2) pathway and the control of the ATF3 (Activating transcription factor 3)-IkBζ (Nuclear factor of kappa light polypeptide gene enhancer in B-cells inhibitor zeta) (110, 111). The last break of TCA cycle in response to LPS occurs at the level of succinate. Succinate production is highly induced after LPS treatment in a glutamine dependent manner, which stabilizes HIF-1α and the expression of IL1β (104). Succinate can also promote this pathway through the succinylation of PKM2 by SIRT5. Once PKM2 is succinylated it can form a heterodimer with HIF1α and promote the expression of IL1β (112). Another mechanism by which succinate can activate HIF1α is through its oxidation, coupled with an increased mitochondrial potential membrane, which increases ROS production and thus the stability of HIF1α (113). Interestingly, succinate can also promote inflammation through an autocrine and a paracrine manner *via* its release into the extracellular milieu and its sensing by the receptor GPR91 (114).

The role of fatty acid in macrophages treated by LPS is poorly understood. While the FAO seems to not be important for macrophage polarization, the fatty acid synthesis might play a role. In fact, LPS-treated macrophages increased their production of triglycerides which is associated with an increase in CD36 expression (115). The production of FA is regulated through a UCP2 (Uncoupling Protein 2)-FASN (Fatty acid synthase) axis which can trigger the activation of the NLRP3 inflammasome and the production of IL1β and IL18. The regulation of this pathway improves the survival in a model of polymicrobial sepsis (116). *Salmonella* infection promotes the expression of PPARδ in macrophages. This will induce a switch from a glycolytic metabolism toward FA metabolism in host cells and allow *Salmonella* to use the available glucose to promote its replication (117). *Mycobacterium tuberculosis*, through an IFNγ-HIF1α axis, promotes the formation of lipid droplets. Lipid droplets are not used by the bacteria for replication, but are rather used by macrophages to promote the production of PGE2 and LXB4 (Lipoxin B4) to support host defense (118). Live *Mycobacterium tuberculosis* (in contrast the dead or attenuated bacteria) also shifted the mitochondrial and glycolytic metabolism toward quiescence and induces a higher dependency of mitochondria to use exogenous fatty acid as a source of energy (119).

Amino acids also have a role in the regulation of macrophages during bacterial infection. Glutamine is a crucial metabolite for the production of NO as well as for IL1β (120, 121). Of note, a similar phenomenon also occurs in macrophages activated by BCG (122). Glutamine can feed the TCA cycle and is notably responsible for the increase of succinate observed after LPS treatment by inducing a GABA shunt. Inhibiting this glutamine-induced GABA shunt protects mice against LPS-induced sepsis and *S. Typhymurium* infection in mice (104). While the role of this metabolite is less described, serine is required for the optimal expression of IL1β gene and the blockade of *de novo* serine synthesis improve survival in a model of LPS-induced sepsis (123). The role of tryptophan metabolism is more controversial. Studies have demonstrated that LPS or IFNγ can induce the expression of IDO and the degradation of tryptophan could have an anti-bacterial effect (47). However, another group reported that IL4L1 can block the LPS effect in macrophages through induction of tryptophan catabolism, suggesting that its exact role needs to be further studied (76). Finally, the arginine metabolism is another crucial pathway to modulate the anti-bacterial response by regulating the balance between citrulline/NO (notably *via* iNOS) and the levels of ornithine and urea (notably *via* ARG1) (38, 124). It also appears that *Mycobacterium tuberculosis* regulates several amino acid transporters and metabolic enzymes but the exact mechanisms by which it affects the host response versus the bacterial survival remains to be elucidated (125).

Despite the well described effect of LPS, other bacterial infections have been described to modulate the host metabolism. LPS has been widely used to study macrophage immunometabolism because it is a simple way to mimic bacterial infections and it shares functional similarities with other TLR ligands. However, it is a single product of gram-negative bacteria (and therefore does not mimic the possible effect of gram-positive bacteria) and it does not mimic the complex *in vivo* settings in which several TLR ligands and inflammatory mediators, as well as several types of bacteria, can modulate immunometabolism. Besides LPS, infection of human macrophages with *Legionella Pneumophila* induces aerobic glycolysis and the inhibition of glycolysis by 2-DG reduces bacterial replication. The OXPHOS is however largely suppressed due to mitochondrial fragmentation through accumulation of DNM1L (Dynamin 1 like) (126, 127). *L. Pneumophila* also induces the production of itaconate by IRG1 which promotes the bacterial clearance as a host protective mechanism (128). Besides these effects, *L. Pneumophila* modulates the expression of genes involved in lipid and amino acid metabolism but more studies are needed to precisely define the roles of these genes (129). During *Mycobacterium tuberculosis* infection, interstitial and alveolar macrophages both have different roles and metabolism. In mice, interstitial macrophages use the glycolysis to differentiate toward a pro-inflammatory phenotype and control bacterial growth as well as the mice survival. On the other hand, alveolar macrophages use the fatty acid oxidation and are not able to control bacterial infection. Blocking glycolysis with 2-DG enhances bacterial replication while blocking FAO with etomoxir decreases bacterial replication (130–132). Interestingly, the control of glycolysis in alveolar macrophages appear to be dependent on miR-21 which controls the expression of PFKM and IL1β (133). Finally, and similarly to other pathogens, *Staphylococcus aureus* has to ability to modulate the host metabolism which has been recently reviewed recently (134–137). Another important metabolic regulation of bacterial infection through metabolism is that bacteria can reroute the macrophage metabolism to use nutrients for their own use which is nicely described elsewhere and might be a key in the macrophage response to infections (138–141).

## Role of Macrophage Immunometabolism During Viral Infection

During viral infections, macrophages will elicit a pro-inflammatory response similar to what have been described during bacterial infection. Coupled to this, macrophages will also start producing type I interferons (interferon alpha and beta). The sensing of ssRNA, dsRNA and unmethylated DNA with CpG motifs *via* TLR3, 7 and 9, respectively, will trigger type I interferon production by macrophages. The RLR family members will also recognize viral motifs and mediate the production of type I interferons (142). Type I interferons will therefore signal through their receptors (Interferon-α/β receptor 1 and 2) which will lead to the activation of the PI3K, MAPKs, STATs and IRF9 ultimately leading to the induction of the ISGs (Interferon Stimulated Genes). Theses ISGs include genes implicated in the mount of antiviral responses, inflammation, pro- and anti-apoptotic molecules as well as regulation of translation and RNA turnover. Type I interferons can be produced by a broad range of cells including macrophages, dendritic cells, epithelial cells, fibroblasts, as well as plasmacytoid dendritic cells (which is the primary source of interferons during viral infection). Type I interferon will therefore signal in the abovementioned cells as well as in T and B cells (143, 144). Interestingly, while macrophages were not supposed to be the primarily source of type I interferon producers, it clearly appears that they are able to control viral infection through production of IFNα/β and are among the first responders during viral infections (145, 146).

The exact role of glycolysis during viral infection remains unclear. First of all, the expression of GLUT1 is increased in monocytes from HIV infected patients and is linked to an increase in glucose uptake and the generation of pro-inflammatory monocytes (147) (**Table 1**). HIV-I infection in monocytes/macrophages also promotes the expression of HK1 and its localization to the mitochondria thus protecting the cells from apoptosis (148). However, a previous study reported that HIV-I infection of macrophages decreased the glucose uptake and the levels of several glycolytic intermediate (149). These discrepancies suggest that the timeline of infection as well as other parameters like cell differentiation status (monocytes vs macrophages) and the phenotype (pro- vs anti-inflammatory) might play a part for the regulation of glycolysis into HIV-infected macrophages and need to be further studied (150). During Dengue virus infection, the expression of GLUT1 and HK2 are increased as well as the level of early (from glucose to glyceraldehyde 3 phosphate) glycolytic metabolites (G6P, F6P), while the levels of late (from glyceraldehyde 3 phosphate to pyruvate) glycolytic metabolites is increased at shorter time (10 h) and decreased later (48 h). Interestingly, glycolysis supports the viral replication and inhibition of glycolysis using oxamate (a competitive inhibitor of LDH) and 2-DG blocks the viral replication (151). A similar phenomenon occurs during murine norovirus infection (152). While the authors of these two papers did not study further mechanisms, they hypothesized that an increase in glycolysis could promote the generation of biomolecules needed for their replication such as lipids, ATP or NADH. The role of glycolysis during viral infection might be virus dependent. In fact, during VSV (Vesicular stomatitis virus) infection, glycolysis is increased through a type I IFN dependent pathway. The expression of several glycolytic enzymes is increased in this context, and more particularly the expression of PFKFB3 which supports the viral phagocytosis and protects the mice during RSV (Respiratory syncytial virus) infection *in vivo* (153). Finally, during SARS-CoV2 infection, macrophages largely increase their glycolytic levels which promotes the viral replication and the production of pro-inflammatory cytokines. Mechanistically, the infection induces the production of mROS leading to the stabilization of HIF1α, thus promoting glycolysis. Interestingly, these changes in metabolism inhibit T cell responses and reduce epithelial cell survival (154).

Little is known regarding the role of PPP during viral infections in macrophages. A pioneer study underlined that 6PG and S7P are decreased during HIV-1 infection and the ratio NADP/NADPH is largely decreased (149). However, the functional role of these changes in PPP remains unknown and will have to be further studied. Importantly, it has to be noted that virus, similarly to what has been described above for bacteria, can hijack the host glycolysis (and metabolism in general) in an attempt to use these nutrients to sustain their replication and survival in the host (155, 156).

Similarly, the function of TCA cycle is poorly defined. During HIV infection, the levels of the TCA cycle metabolites are unchanged (except for malate which is increased) (149). However, macrophages surviving HIV infection present an altered TCA cycle and OXPHOS (157).

On the other hand, the activation of macrophages by viruses and their products largely remodels their lipid pool. The stimulation of macrophages with TLR3, 7 and 9 agonists to mimic viral infection modulates the lipid composition of nearly all lipid classes. These changes largely occur through a MyD88 and TRIF signaling pathway and the interferon signaling is also a requirement to these changes. Interestingly, the TLR3 and TLR7/9 signaling differentially modulate the fatty acid synthesis respectively because of their use of the TRIF (TIR-domain-containing adapter-inducing interferon-β) and MyD88 (Myeloid differentiation primary response 88) signaling pathways. While TLR3 stimulation decreased the generation of saturated long chain fatty acid (SFA) and monounsaturated long chain fatty acid (MUFA), the TLR7/9 stimulations increased these levels. Mechanistically, the MyD88-NRF2 (Nuclear factor erythroid 2-related factor 2)/SREBP (Sterol regulatory element-binding protein) axis induces the expression of stearoyl-CoA desaturases 1 and 2 which negatively controls the inflammation (*Il1b*, *Il6* and *Cxcl1* expression notably) through an increased production of these MUFAs (158). While this study was performed using only TLR agonists, other publications in different cell types suggest that targeting fatty acid during different types of viral infections might be a strategy to control viral replication (159–161). Type I interferons can also promote the import of cholesterol and long chain fatty acid during murine gammaherpesvirus-68 (MHV-68) infection (but also with HIV). In this setting, blocking the lipid import protects from viral infection through production of type I interferon in a STING-dependent manner (162). Moreover, the infection of macrophages by HIV impairs the cholesterol efflux through a Nef (Negative regulatory factor)-ABCA1 (ATP-binding cassette A1) pathway inducing the formation of foam cells. In the meantime, the activation of TLR8 in macrophages by ssRNA from HIV reinforces this foam cell phenotype through the production of TNFα (163, 164). Besides these effects, the cholesterol has also largely been demonstrated to be crucial for the virus entry in the cell and the anti-viral response (165–168). A growing number of evidence showed that SARS-CoV2 infection is linked to a reprogramming in lipid metabolism with cholesterol playing a crucial role. Indeed, membranes rich in cholesterol are a point of entry of SARS-CoV2 in the cells (169). The enzyme cholesterol 25-hydroxylase (CH25H, belonging to the ISGs) is highly induced during SARS-CoV2 infection and restricts viral infection by depleting cholesterol on the plasma membrane (170, 171). Moreover, SARS-CoV2 promotes the expression of several lipid synthesis modulators (including SREBP1/2, CD36, PPARγ or DGAT-1) leading to the production of cholesterol and lipid droplets. Blockade of this pathway can decrease both the viral replication and the inflammatory response induced by SARS-CoV2 (172, 173).

The modulation of amino acid uptake and production is also a crucial regulator during viral infections. The amino acid glutamine is the main source of energy during HIV latent infection along with glutamate and α-ketoglutarate and blocking the use of these metabolites induces the death of latency infected macrophages (157). EBV (Epstein-Barr virus) and HIV infections both induce the expression of IDO in macrophages in an IL6 and TNFα dependent manner thus inhibiting the activation of T cell activation. Inhibition of IDO eventually lead to the elimination of the macrophages infected by viruses (174, 175). The arginine metabolism is regulated in a complex manner during viral infection. The activation of innate immune responses by viruses induces the production of NO by macrophages and other immune cells (176). But some viruses like the Sendai virus will try to limit the production of NO as a mechanism to escape host responses (177). In fact, while beneficial at first, a sustained production of NO over the time will lead to damage the host tissues and inhibits the Th1 responses (176); (178). Arginine, in the other hand, is a critical metabolite for the replication of viruses and the inhibition of Arg1 reduces the viral replication and ability to infect the host cells (179). However, Arg1 might also promote the tissue repair after viral infection [for an extensive review about the role of arginine metabolism see (180)]. Finally, viruses can target mTOR to modulate the innate immune responses. For example, Vaccinia virus encode the protein F17 which as the ability to disrupt the mTOR complex in the Golgi which will block the activation of STING (Stimulator of interferon genes) and the generation of an interferon-mediated immune response (181, 182). Additionally, viruses can modulate mTORC1 to inhibit host protein translation or promote the translation of their own mRNAs (183–185).

## Role of Macrophage Immunometabolism During Trained Immunity

The immune system is classically divided into two arms: the innate immune system and the adaptive immune system. Scientists assumed for a long time that only the adaptive immune system has an immunological memory, and that the innate immune system was only able to sense pathogens in a partially unspecific manner (through the PRR) which does not last over the time. However, this concept has recently been largely challenged. In fact, it appears that innate immune cells do have an ability to develop a broad immunological memory that lasts over the time (and could even be antigen-specific in some cases) (186, 187). These memory-like responses are now well known as trained immunity (188–190). Overall, trained immunity will induce an enhanced inflammatory response in response to secondary stimuli marked by the increased ability of monocytes to produce inflammatory cytokines (notably TNFα and IL6) trough sustained changes in metabolism. In human monocytes, the stimulation with β-glucan followed by 7 days of resting period leads to a decreased level of OXPHOS, increased glucose consumption and lactate production (**Table 1**). An AKT-mTOR-HIF1α pathway is responsible for this increase in glycolysis and its blocking (either by pharmacological or genetic inhibition) abrogates this trained immunity and is protective in lethal models of *C. albicans* and *S. aureus* infections (191). Glutaminolysis is also required for the induction of trained immunity through its ability to sustain the production of fumarate which will modulate the stability of HIF1α and KDM5 (Lysine deacetylase 5) activity, thus promoting the epigenetic reprogramming at the promoter of IL6 and TNFα (192). The cholesterol pathway is also linked to the induction of trained immunity through the induction of mevalonate production (192, 193). Mechanistically, mevalonate promotes the function of IGF1R (Insulin-like growth factor 1 receptor) and mTOR leading to subsequent epigenetic changes (193). A similar glycolysis-AKT-mTOR-epigenetic pathway is also involved during BCG (Bacille Calmette-Guerin)-induced trained immunity demonstrating that this pathway might be a general process during different type of stimuli inducing trained immunity (194). Interestingly, a similar phenomenon is observed in hematopoietic myeloid progenitors and increases the myelopoiesis (195). At the opposite of the concept of trained immunity is the concept of immune tolerance (or immunoparalysis) which induced a persistent tolerance in macrophages over the time, notably in response to LPS (186, 189). Tolerant monocytes from sepsis patients show an impair levels of OXPHOS and glycolysis and IFNγ can restore the metabolic defects through activation of mTOR (196). The state of tolerance is induced by itaconate and β-glucan can revert this state of tolerance by blocking the expression of Irg1 and increasing the expression of Sdh eventually reversing the immunoparalysis (197, 198).

## Targeting Macrophage Immunometabolism as a Potential Therapeutic Target for Infections: an Emphasis on COVID-19

Macrophages have, for a long time, be considered potential targets to control immune responses in a wide variety of diseases (199, 200). As new research shed light on the role of immunometabolism in macrophages, it becomes clear that targeting the immunometabolism in macrophages can be a therapeutic target during the development of many diseases including infections. A current focus of research in the past few months has been the development of drugs and vaccines for the SARS-CoV-2 (Severe acute respiratory syndrome coronavirus 2) infection that leads to the development of COVID-19 (Coronavirus disease 2019) and firstly appeared in December 2019 in Wuhan, China. In fact, the development of potential therapeutics is critically needed since it already affected more than two hundred fifty million people worldwide and led to 5,284,432 deaths (according to the daily WHO report on December 7^th^). As other viral infections, COVID-19 induces the development of an immune response in which the innate immune cells (and notably macrophages) are the first line of defense (201). Several studies have been reported that the progression to severe forms of infection by COVID-19 (but this also true for many viral and bacterial infections like the development of sepsis) is associated with an overt and dysregulated production of inflammatory factors like IL1β, IL6, TNFα, IFNγ, GMCSF, CCL2, CCL3, CCL4, CXCL10 and many others (202, 203). This cytokine release syndrome (or cytokine storm) is responsible for damages during infections and more particularly into the lungs of patients of sepsis and COVID-19 [also called Acute respiratory distress syndrome (ARDS)] and plays a major role in the related deaths observed in patients with severe conditions (204–207). Importantly, the monocyte/macrophage system is largely remodeled during acute SARS-CoV2 infection with an increased proportion of inflammatory monocyte infiltration in patients with severe condition and macrophages harboring a highly pro-inflammatory phenotype (208, 209). Interestingly, a similar phenomenon occurs during SARS-CoV infection (210, 211). As discussed before, many of these parameters are regulated by metabolism suggesting that targeting metabolism might be a therapeutic strategy to protect against this overt inflammation during severe infections and more particularly during COVID-19. Five drugs targeting metabolism are currently in use clinically to treat different diseases and could be used to prevent the cytokine storm: dimethylfumarate (DMF), metformin, methotrexate, rapamycin and dexamethasone (**Table 2**).

**Table 2:** Potential therapeutic molecules for the treatment of infections.

Molecule	Target	Consequence
Already used in clinic		
DMF	NRF2-KEAP1, NFkB, ERK, GAPDH	Decreases glycolysis and inflammation, promotes an anti-inflammatory phenotype.
Metformin	Complex I of OXPHOS	Inhibits ROS, ATP and IL1 β production, promotes IL-10 production.
Methotrexate	AICAR (at low dose)	Raises adenosine levels and activates AMPK. Decreases IL1β, IL6 and TNFα levels.
Rapamycin	mTOR	Promotes tolerance and controls glycolysis and inflammation.
Dexamethasone	Multiple possible targets (including mTOR, NFkB…)	Promotes tolerance. Increases OXPHOS and ROS levels. Antibacterial effect.
In development		
2-DG	Hexokinase	Blocking of glycolysis. Decreases inflammatory responses.
TEPP-46	PKM2	Inhibits glycolysis, HIF1α and IL1β production
DMM	SDH	Inhibits HIF1α and IL1β production, promotes IL-10 and IL1RA production.

2-DG, 2-deoxyglucose; Aa, Amino acid; AICAR, Amido-imidazolecarbox-amido-ribonucleotide; DMF, Dimethylfumarate; DMM, Dimethylmalonate; Erk, Extracellular-signal-regulated kinase; FAO, Fatty acid oxidation; FAS, Fatty acid synthesis; HIF1α, Hypoxia factor 1 alpha; HK, Hexokinase; HMG-CoA, β-hydroxy b-methylglutaryl-CoA; IL, Interleukin; KEAP1, Kelch-like ECH-associated protein 1; mTOR, mammalian target of rapamycin; NFkB, Nuclear factor kappa B; NRF2, Nuclear factor erythroid 2-related factor 2; OXPHOS, Oxidative phosphorylation; PKM, Pyruvate kinase muscle isotype; ROS, Reactive oxygen species; SDH, Succinate dehydrogenase; TNFα, Tumor necrosis factor alpha.

Firstly, DMF (a fumarate analog and NRF2 activator currently used for the treatment of multiple sclerosis) notably inhibits NFkB, ERK (Extracellular-signal-regulated kinase) and other signaling pathway. In macrophages, DMF will notably activates NRF2 to protect the cells from oxidative stress and promote an anti-inflammatory phenotype (212). DMF also acts on glycolysis since it decreases the activity of GAPDH suggesting therefore that DMF could be repurposed to modulate immunometabolism during infectious diseases (213). Similarly to DMF, 4-OI (an itaconate analog not used in clinic for the moment) target the NRF2-KEAP1 pathway and might prevent the cytokine storm during acute infections (214). These 2 analogs work by mimicking their respective metabolites suggesting that fumarate and itaconate modulation could be interesting targets to dampen inflammation in infectious settings. Interestingly, a recent report suggested that these 2 drugs might have a potent antiviral and anti-inflammatory activity during COVID-19 (DOI:10.21203/rs.3.rs-31855/v1, under review in Virology).

Metformin is a first-line treatment for the treatment of type 2 diabetes and metabolic disorders notably through a glucose lowering effect. Interestingly, metformin has also an immunomodulatory function. Both these functions depends on the ability of metformin to activate AMPK. This occurs through the ability of metformin to inhibits the complex I of ETC which controls the production of ATP and ROS. The blockade of ATP generation will lead to an increase in AMP or ADP/ATP ratio which will consequently activate AMPK (215). Metformin is known to suppress the production of IL1β and promotes the production of IL10 in response to LPS (216). Interestingly, metformin has been used in the 1940’s as an antimalarial drug as well as to treat influenza and might show interesting properties in the treatment of *M. tuberculosis* and COVID-19 (217–220). Importantly, three recent studies have suggested that metformin could be used as a therapeutic during HIV, SARS-CoV2 and *Mtb* infections (219, 221–223). In all pathologies, metformin use has been linked to an improved survival in diabetes and obese patients. Mechanistically, metformin reprograms the immunometabolism of CD4 and CD8 T cells which lead to a modulation of viral replication and enhanced the immune responses. However, whether macrophages can be targeted by metformin remains to be studied (219, 223). Metformin has therefore be proposed to possibly be a treatment for several bacterial, protozoal and viral infections (https://doi.org/10.1002/dmrr.2975), and several clinical trials to assess the use of metformin during HIV (NCT04500678, NCT02383563, NCT02659306, NCT04930744) Mtb (NCT04930744) or SARS-CoV2 (NCT04510194) infections are now ongoing. However, despite a relative good safety profile, metformin is associated with several side effects (notably at the cutaneous and gastro-intestinal tract levels) and approximately 5% of the patients have to discontinue the treatment (224).

At high dose, the methotrexate is an inhibitor of the DHFR (Dihydrofolase reductase) which will block the downstream inhibitors of the folate pathway eventually leading to the inhibition of nucleotide synthesis. At lower dose, methotrexate is inhibiting AICAR (Amido-imidazolecarbox-amido-ribonucleotide) leading to the increased production of the anti-inflammatory factor adenosine. Methotrexate can induce the activation of AMPK and further inhibit the production of IL1β, IL6 and TNFα in macrophages in response to LPS (and can also inhibit the activation of pro-inflammatory B and T cells and promote the generation of Treg) suggesting a potential therapeutic efficacy to control the overt inflammation during SARS-CoV2 infection (225–227). The effect of methotrexate on viral-induced inflammation has been or is currently being tested in two cohorts of SARS-CoV2 and HIV patients and will require further investigations (NCT01949116, NCT04352465). Methotrexate is currently used as an anti-tumoral, anti-psoriatic and anti-arthritic drug. However, due to its immunomodulatory effects, methotrexate is associated to an increased level of infection in rheumatoid arthritis patients. Moreover, its use is also linked to hepatotoxicity, pulmonary toxicity, nephrotoxicity, hematologic toxicity as well as gastrointestinal side effects and carcinogenicity and 20-30% of patients have to stop the usage of this drug due to these side effects (which could remain for up to 5 years) therefore emphasizing the need of more research to determine its potential use to treat infections (228).

Another interesting target is to modulate mTOR by using the inhibitor rapamycin (or similar mTOR inhibitors like everolimus, vistusertib or AZD8055). It has been demonstrated that rapamycin can protect mice against inflammation and death (through control of macrophages) in a model of CLP-induced sepsis (229). Moreover, derivatives of rapamycin were shown to reduce the rate of infection to influenza in elderly in without side effects (230). These findings have led to the hypothesis that rapamycin might be a potential target to treat infections and might be of great interest in severe forms of COVID-19 (231, 232). mTOR has also been hypothesized to be a therapeutic target during *Mtb*, T cruzi or HIV infections (233–235) and the safety and efficacy of sirolimus is currently being tested in a clinical trial as a Covid-19 treatment (NCT04461340). As an immunosuppressant, blocking mTOR (notably with the use of sirolimus) is linked to development of cancer (especially lymphoma and skin cancer), infections and other adverse events including hyperglycemia and dyslipidemia (236).

Finally, Dexamethasone, a synthetic glucocorticoid with anti-inflammatory and immunosuppressive properties is widely used to treat inflammatory conditions. Dexamethasone acts largely through macrophages by decreasing their production of pro-inflammatory factors (like CCL2, TNFα, COX-2…) (237–239). Dexamethasone is able to promote bacterial phagocytosis and killing by human macrophages *in vitro* and is protective in a model of LPS-sepsis (240, 241). A possible mechanism of action is also to increase the expression of OXPHOS genes and to promote the production of ROS by macrophages finally leading to suppress the T cell responses (242, 243). Interestingly, dexamethasone has recently been determined to be the first drug to save lives in the SARS-CoV2 infection. The RECOVERY trial enrolled 2100 patients treated with low to intermediate doses of dexamethasone (6 mg per day for 10 days) compared to patients receiving standard care. The survival rate was improved by 30% in patients receiving invasive ventilation and by 20% in patients receiving oxygen support (without invasive mechanical ventilation) (244). However, as a corticosteroid with an immunosuppressive action, dexamethasone has been described to have several side effects and the dose used appears to be critical (245).

Besides the drugs already approved in clinic, the development of drugs inhibiting the metabolites implied in the mounting of an immune response can be targeted as well (**Table 2**). For example, the development of 2-DG (HK2 inhibitor, currently tested in 219 clinical trials currently), TEPP-46 (PKM2 activator) or dimethylmalonate (DMM, SDH inhibitor, tested in phase 2 clinical trials currently) might be an important advance in the development of immunometabolic inhibitors (226). These finding provide a crucial understanding on how using drugs targeting macrophage immunometabolism (notably to prevent the cytokine storm) might be used as therapeutic targets during infections and more specifically during SARS-CoV2 Infection.

## Concluding Remarks

The past decade has seen a great development in our understanding on how the metabolism can regulate immune responses. Macrophages have been demonstrated to be a key player in how immunometabolism regulates the mount of a proper immune response during different types of infections. Although the modulation of metabolism has been largely described *in vitro* in response to LPS and IL4, its role in different complex microenvironment and more importantly *in vivo* remains largely poorly understood. Moreover, much of the data has been published in mice and the potential to target metabolism in humans must be further studied. While the different major metabolic pathways are seen as a unique block, they all intersect each other and a break in a unique metabolite might largely affect the cell behavior rendering the things more complex. Despite these limitations, the possibility to target metabolism in macrophages to control infectious disease has shown a great potential and might play an important role in the finding for a cure of different infections, including COVID-19. Indeed, the modulation of macrophages phenotype is a promising target since, contrary to many other immune cell types, it can be targeted in a specific manner through the use of liposomes or other cellular “backpack”, thus limiting specificity and side effects (246, 247). Based on these facts, it is likely that an increase in macrophage immunometabolism understanding will provide new insights to cure infections.

## Author Contributions

TG and WC drafted and edited the manuscript. All authors contributed to the article and approved the submitted version.

## Funding

This research was supported by the Intramural Research Program of NIDCR.

## Conflict of Interest

The authors declare that the research was conducted in the absence of any commercial or financial relationships that could be construed as a potential conflict of interest.

## Publisher’s Note

All claims expressed in this article are solely those of the authors and do not necessarily represent those of their affiliated organizations, or those of the publisher, the editors and the reviewers. Any product that may be evaluated in this article, or claim that may be made by its manufacturer, is not guaranteed or endorsed by the publisher.
